# A blind passenger: a rare case of documented seroconversion in an *Angiostrongylus cantonensis* induced eosinophilic meningitis in a traveler visiting friends and relatives

**DOI:** 10.1186/s40794-019-0084-x

**Published:** 2019-04-15

**Authors:** Tobias Brummaier, Sonja Bertschy, Kornelius Arn, Thomas Treumann, Marie-Therese Ruf, Beatrice Nickel, Daniel H. Paris, Andreas Neumayr, Johannes Blum

**Affiliations:** 1 Department of Medicine, Swiss Tropical and Public Health Institute, Basel, Switzerland; 2 Faculty of Medicine, University of Basel, Basel, Switzerland; 3 Shoklo Malaria Research Unit, Mahidol-Oxford Tropical Medicine Research Unit, Faculty of Tropical Medicine, Mahidol University, Mae Sot, Thailand; 4 Centre for Tropical Medicine and Global Health, Nuffield Department of Medicine, University of Oxford, Old Road Campus, Oxford, UK; 5 Department of Infectiology, Luzerner Kantonsspital, Luzern, Switzerland; 6 Division of Hematology and Hematology Laboratory, Luzerner Kantonsspital, Luzern, Switzerland; 7 Division of Nuclear Medicine and Radiology, Luzerner Kantonsspital, Luzern, Switzerland

**Keywords:** *Angiostrongylus cantonensis*, Eosinophilic meningitis, Seroconversion, Switzerland, Visiting friends and relatives

## Abstract

**Background:**

Eosinophilic meningitis (EOM) is a rare condition that is caused by various communicable and non-communicable factors. The rat-lungworm *Angiostrongylus cantonensis*, which is associated with consumption of raw or undercooked paratenic or intermediate hosts, is the most common cause of parasitic eosinophilic meningitis worldwide. While the majority of *A. cantonensis* cases are reported from endemic regions, cases in travelers pose a challenge to clinicians in non-endemic countries. Here we report a rare case of eosinophilic meningitis caused by *A. cantonensis* in a Swiss traveler who was diagnosed after returning from Thailand.

**Case presentation:**

A 33-year old woman with a travel history to rural north-eastern Thailand presented to an emergency department in Switzerland with severe headache and vomiting. Eosinophilic meningitis was confirmed as the cause of the symptoms; however, serologic investigations failed to confirm an *A. cantonensis* infection on the first evaluation. Nevertheless, empirical treatment with an anthelminthic and steroid regimen led to a rapid alleviation of symptoms. Repeated serology confirmed seroconversion 2 weeks after treatment initiation.

**Discussion:**

Parasitic etiology must be considered in returning travelers who present with symptoms compatible with a central nervous system infection. A thorough medical history, including types of food consumed, is paramount and can often suggest differential diagnosis. Neuroangiostrongyliasis is rare and might be missed if serology does not cover possible seroconversion.

## Background

Parasitic central nervous system (CNS) infections are expected to become more common in non-endemic countries. Parasitic etiologies require more consideration in travelers presenting with symptoms suggestive of a CNS infection. Overlapping and often non-specific clinical symptoms in combination with their scarcity and epidemiologic peculiarities make parasitic CNS infections a diagnostic challenge [1]. The rat-lungworm *Angiostrongylus cantonensis* is the most common cause of parasitic eosinophilic meningitis (EOM) worldwide [2]. Infective third-stage *Angiostrongylus* larvae are found in intermediate hosts (i.e. gastropods such as snails or slugs), food products contaminated with gastropods or gastropod mucus (e.g. vegetables, fruits) or in paratenic hosts (e.g. shrimp, prawns, crabs, monitor lizards, frogs, mussels). Accidental or deliberate ingestion of raw or undercooked food products containing the neurotropic third-stage larvae causes CNS infections in humans [3]. Larvae pass the gastrointestinal tract, actively cross into systemic circulation and migrate to the central nervous system. Symptoms occur after an incubation period that varies from 7 to 35 days [3].

**Fig. 1:**
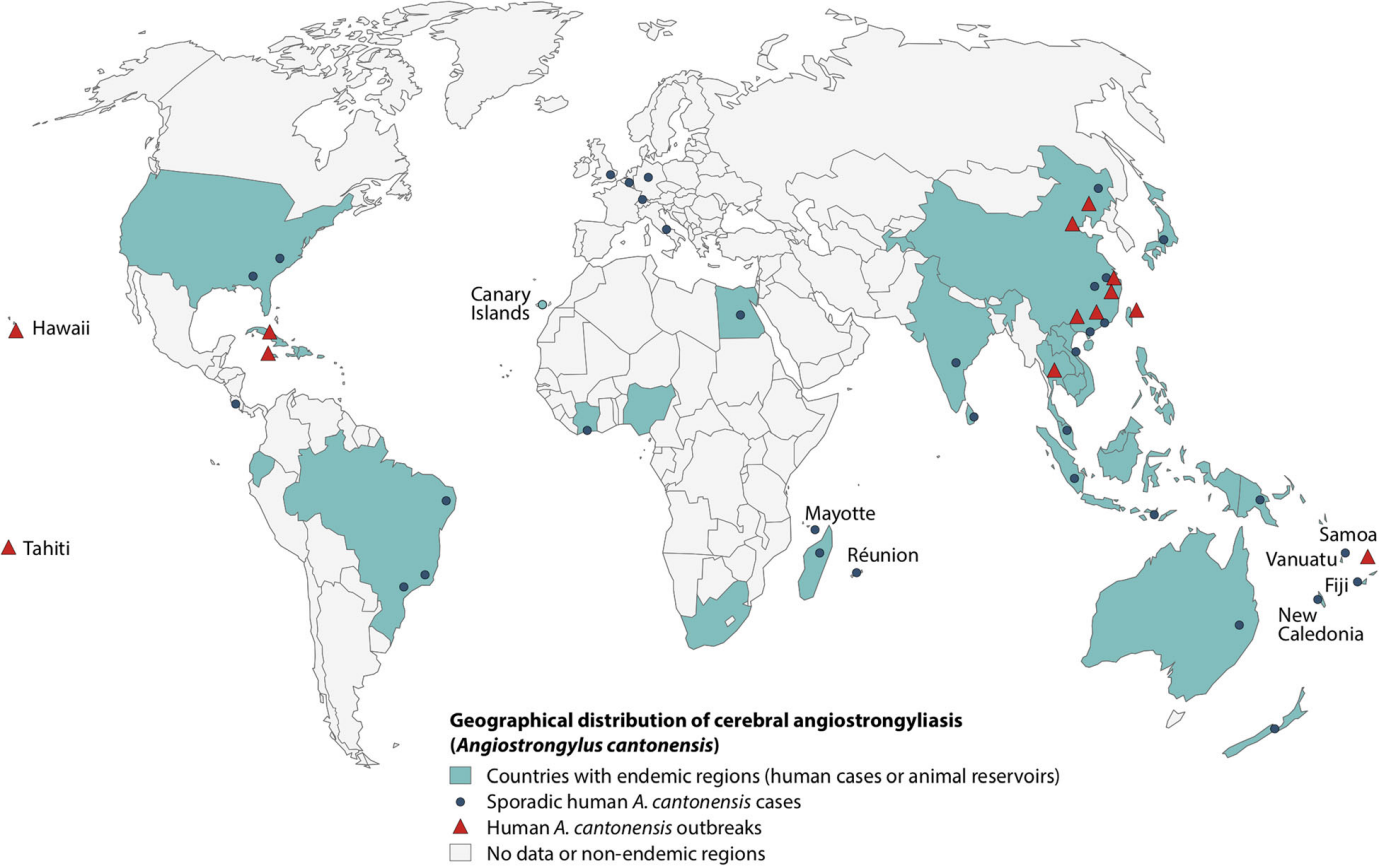
Geographical distribution of cerebral angiostrongyliasis. Legend: Map created by Rosalie Zimmermann, adapted from Wang QP et al. Lancet Infect Dis 2008;8:621–630 and Barratt J et al. Parasitology 2016;143:1087–1118

*A. cantonensis* cases are primarily reported from endemic regions (Fig. 1) [4]. However, due to an increase in international travel, individual tourism, and a change in risk behavior, an increment in sporadic cases of *A. cantonensis* in non-endemic countries is expected [5]. The rare occurrence and usually self-limiting course of the disease are 2 factors that contribute to the assumption that many cases of *A. cantonensis* remain undiagnosed.

Here we present a rare case of an EOM caused by *A. cantonensis* in a visiting friends and relatives (VFR) traveler.

## The case

In August 2018 a 33-year old woman presented with a history of progressive headache, nausea and vomiting. The patient is of Thai origin but has resided in Switzerland for 6 years. However, she regularly travels to rural north-eastern Thailand, which was also the case prior to onset of symptoms. Two weeks after arrival in Thailand, the patient noted self-limiting diarrhea that was followed by non-specific symptoms (i.e. fatigue, feeling of generalized swelling, leg accentuated weakness). Two weeks after onset of these non-specific symptoms the patient returned to Switzerland and reported symptom aggravation in the form of gradually increasing headache. The initial headache location shifted from the right to the left hemisphere and eventually manifested as holocranial headache of dull-throbbing character. The patient, who denied being prone to headaches, described severe headache (numerical rating scale 7–10) that was not responsive to nonsteroidal anti-inflammatory drugs. Insomnia due to persistence of headache with additional occurrence of nausea and vomiting prompted the patient to attend the emergency department 5 days after returning from Thailand.

On admission, the patient was afebrile (temperature 36,8 °C), fully conscious (Glasgow Coma Scale 15) and without focal neurologic deficits or signs of meningism. The medical history revealed a pre-existing, inactive hepatitis B infection and subclinical hypothyroidism. For both conditions no treatment was taken at the time of presentation. From routine screening during a previous pregnancy, HIV was known the be negative. A complete blood count (CBC), clinical chemistry, a lumbar puncture (LP) and cranial computed tomography (CT) was ordered. The CBC indicated a neutrophil predominance and biochemistry results were unremarkable. In the cerebrospinal fluid (CSF) eosinophilia was noted (Table 1), a finding that was not reflected in the peripheral differential count.

**Table 1:** Cerebrospinal fluid and blood results

Test (Unit)	Result	Reference Range^a^
Cerebrospinal fluid		
• Appearance	**Cloudy**	Clear
• Xanthochromia	Negative	Negative
• Erythrocytes (× 10^12^/L)	0	0
• Cell count (× 10^9^/L)	**1067**	0–3
• Lymphocytes (%)	56	
• Monocytes (%)	19	
• Eosinophils (%)	**25**	‡
• Total protein (g/L)	**0.54**	0.15–0.45
• Albumin (g/L)	**0.299**	0.06–0.24
• Glucose (mmol/L)	2.6	2.2–3.9
• Lactate (mmol/L)	1.8	1.2–3.9
Complete Blood Count		
• Leukocytes (×10^9^/L)	6.5	2.6–7.8
o Neutrophils (× 10^9^/L)	**6.21**	0.9–4.5
o Banded neutrophils (×10^9^/L)	0.04	0.0–0.2
o Eosinophils (×10^9^/L)	0.01	0.0–0.4
o Basophils (×10^9^/L)	0.01	0.0–0.05
o Monocytes (×10^9^/L)	0.05	0.0–1.0
o Lymphocytes (×10^9^/L)	0.79	1.0–3.0
• Erythrocytes (×10^12^/L)	4.16	3.7–5.0
• Hemoglobin (g/L)	117	115–148
• Hematocrit	0.35	0.34–0.43
• Platelet (×10^9^/L)	193	130–330
• C reactive protein (mg/L)	< 5	< 5

Abnormal results are shown in bold

^a^Reference ranges according to the hospital laboratory

‡Eosinophilic meningitis is defined as 10 or more eosinophils/μL or eosinophilia of at least 10% of the total CSF leukocyte count [8]

A vascular occlusion in the supply area of the right middle cerebral artery compatible with Moyamoya disease was suspected in the CT scan. Magnetic resonance imaging (MRI) was performed, revealing adequate collateral circulation and no evidence for a recent occlusion or active vasculitis. A few age-appropriate non-specific white-matter lesions were seen in both hemispheres but no leptomeningeal nor nodular enhancing lesions.

**Fig. 2:**
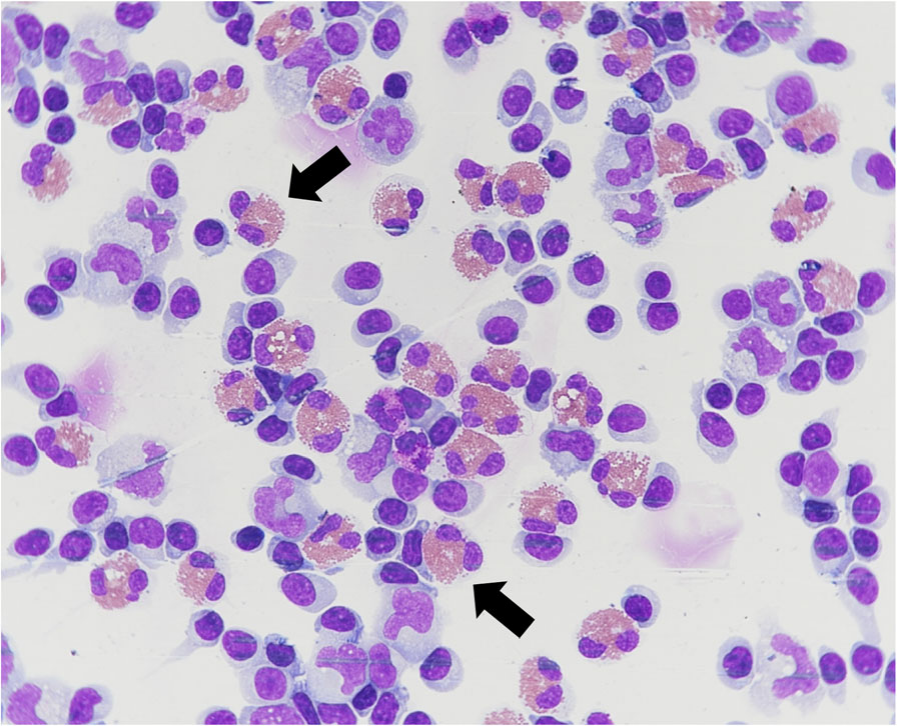
Cerebrospinal fluid smear of our patient. Legend: Pappenheim (May-Grünwald-Giemsa) stain, magnification × 60. Black arrows indicate eosinophil granulocytes with mostly bilobed, occasionally trilobed nucleus and normal eosinophilic granulation

The pleocytosis (cell count of 1067/μL) in the CSF, and the abnormally elevated absolute and relative eosinophil counts of 267/μL and 25% respectively, led to the diagnosis of EOM (Fig. 2). Considering the travel history, an empirical treatment with albendazole (400 mg, BD) and prednisolone (60 mg OD) was started to cover parasitic EOM. Additionally, serological testing for tissue helminths was ordered and performed at the national reference center at Swiss Tropical and Public Health Institute, Basel, Switzerland alongside other neurotropic pathogens.

Enzyme-linked electroimmunotransfer blot (EITB) for *A. cantonensis* and *Gnathostoma spp* specific IgG antibodies performed with serum were nonreactive. IgG screening ELISAs and IgG/IgM/IgA indirect fluorescent antibody tests for *Echinococcus granulosus*, *Fasciola* spp., *Filaria* spp. *Schistosoma* spp., *Strongyloides stercoralis, Trichinella spp* and *Toxocara* spp. as well as screening for other neurotropic pathogens (i.e. *Borrelia burgdorferi*, *Tick-borne encephalitis virus*, *Treponema pallidum*) and HIV were negative. Aside from the afore-mentioned pathogens, the origin of symptoms due to an infection with free living amoeba (FLA) was considered. However, since neither typical features of primary amoebic meningoencephalitis (caused by *Naegleria*) nor granulomatous amoebic encephalitis (caused by *Acanthamoeba*) were seen, no molecular or immunologic tests to rule out a FLA infection were requested.

As acute phase *Angiostrongylus* serology can be negative, the empirical treatment was continued and follow up serology was recommended. The dual anthelminthic and anti-inflammatory therapy led to a marked clinical improvement, and the patient was discharged home after 6 days of hospitalization.

Two weeks after treatment initiation, the patient was seen for a follow up visit in the outpatient department. At this visit, no more symptoms were reported and serologic testing for *A. cantonensis* and *Gnathostoma spp*, the two most likely causes of the initial symptoms were repeated. While the latter remained negative, the serological test for *A. cantonensis* turned positive, indicating seroconversion and, thus confirming the *Angiostrongylus*-associated EOM. Recommended treatment with albendazole (3 weeks) and prednisolone (2 weeks) was completed and the patient recovered completely.

## Discussion

Human health and travelers’ health in particular is strongly influenced by altering interrelationships between international travel, globalization of trade and agriculture, migration as well as food production in a “warming world” [6]. In addition, ripple effects of adventure travel, exotic eating habits and exotic pet trade have repercussions on the epidemiology of communicable diseases that are traditionally considered tropical diseases [6].

In parasitic CNS infections, signs and symptoms are often non-specific and/or overlapping making clinical diagnosis arduous. Travelers returning from potentially endemic countries complaining about severe headache should be generously evaluated for parasitic CNS infections. A thorough travel history and assessment of risk behavior while traveling can guide diagnosis and direct further diagnostic measures. This patient ingested uncooked freshwater mussels from a lake in rural north-eastern Thailand 20 days before the headache commenced.

Magnetic resonance imaging is the radiologic method of choice if parasitic CNS infections are suspected [7]. However, findings in neuroimaging studies have their limitations and also did not contribute significantly to the confirmation of the diagnosis in this patient.

**Fig. 3:**
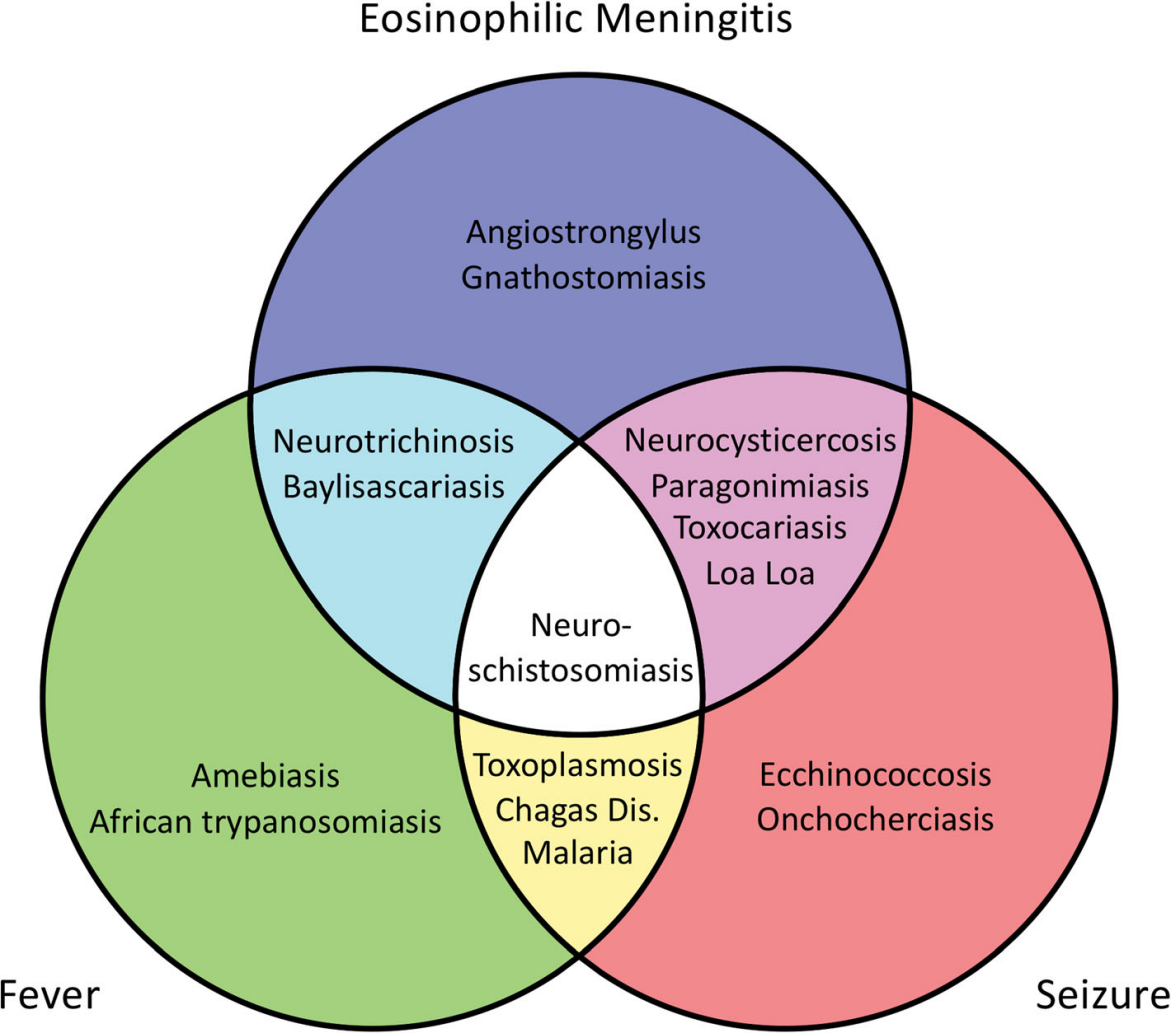
Predominant symptoms in parasitic CNS infections. Legend: Adapted from Carpio et al., Expert Rev. Neurother. 2016;16 (4):401–4,142,016

Confirmatory diagnosis is based on laboratory methods [1]. CSF investigations are paramount in the workup if meningitis is suspected to confirm EOM (i.e. an eosinophil count of ≥10 eosinophils per microliter, or eosinophilia of ≥10% of the total CSF leukocyte count [8]). Various parasitic causes of EOM have to be considered and ruled out where applicable (Fig. 3). CSF xanthochromia is an indicator for the distinction between *A. cantonensis* and *Gnathostomiasis* as it is suggestive of the latter [9]. Absent xanthochromia led to presumptive diagnosis of *A. cantonensis* in the presented case and guided the decision-making process for empirical treatment.

Most cases manifest symptoms in the first 1–2 weeks after larva ingestion. However, incubation periods of months [10] and autochthonous *A. cantonensis* cases from non-endemic areas have been reported [11, 12]. EOM or meningoencephalitis are the most common manifestations followed by ocular angiostrongyliasis [5, 13]. The cardinal symptom in EOM is headache and affects up to 95% of cases [3]. Vasodilatation in the subarachnoid space resulting from widespread inflammatory reaction in the meninges and decreased resorption of CSF lead to increased intracranial pressure, explaining other neurologic findings (e.g. neck stiffness, paresthesia, cerebral nerve palsy, nausea and vomiting) associated with neuroangiostrongyliasis [9, 14].

Since larva recovery from CSF is rare, confirmation is mainly based on detection of antibodies in serum or CSF. Unfortunately, there was not enough residual CSF to perform serologic tests in the patient presented here. Detection of *A. cantonensis* DNA by polymerase chain reaction in the CSF has been described, but is only available in specialized laboratories [2]. As demonstrated in this case, false negative results are possible if serologic testing is restricted to single admission samples (impeding documented seroconversion). Paired serologic testing in weekly time intervals increases diagnostic detection as seroconversion can be rare: only 2 cases of documented seroconversion in human neuroangiostrongyliasis were identified in a systematic literature search [12, 15].

The course of disease is determined by the parasite’s biology in the human host. Humans are considered *dead-end* hosts as the larvae cannot develop to adult worms and die after completed migration to the CNS. This explains the self-limiting course of disease [3, 5] and why most infections are mild. However, prolonged and severe courses as well as fatal cases have been reported [16, 17]. Repeated lumbar punctures are an effective means to relieve headaches by reducing intracranial pressure [9]. Administration of steroids can reduce frequency of lumbar punctures, duration of headache and analgesics use [18]. Anthelmintic drugs are effective against early larval stages [19] and may reduce duration of headache [20]. However, their role in the treatment of neuroangiostrongyliasis remains controversial as they potentially exacerbate neurological symptoms via inducing host immune-inflammatory responses [3, 21]. Consequently, the recommended treatment regimens for *A. cantonensis* are still debated. Some studies found that combinations of antihelminths and steroids are effective [22–24] or at least not inferior and safe [25] in the treatment of EOM caused by *A. cantonensis*. In our case, a combination of albendazole with prednisolone was effective and resulted in a complete recovery.

## Conclusion

Ecological, biological and economic changes can exert their effects on the epidemiology of communicable diseases. Health care providers are increasingly confronted with “exotic” diseases in a merging and globalized world. Even though EOM is rare, preemptive treatment with subsequent follow-up serology is advised if neuroangiostrongyliasis is suspected. Early diagnosis and appropriate management results in favorable outcomes in the majority of cases.

## Abbreviations

BD: Twice per day
CBC: Complete blood count
CNS: Central nervous system
CSF: Cerebrospinal fluid
CT: Computed tomography
EITB: Enzyme-linked electroimmunotransfer blot
EOM: Eosinophilic meningitis
LP: Lumbar puncture
MRI: Magnetic resonance imaging
OD: Once per day
VFR: Visiting friends and relatives
